# New Synthetic Operon Vectors for Expressing Multiple Proteins in the *Chlamydomonas reinhardtii* Chloroplast

**DOI:** 10.3390/genes14020368

**Published:** 2023-01-31

**Authors:** Jihye Yeon, Stephen M. Miller, Wipawee Dejtisakdi

**Affiliations:** 1 Department of Biological Sciences, University of Maryland Baltimore County, Baltimore, MD 21250, USA; 2 Department of Biology, School of Science, King Mongkut’s Institute of Technology Ladkrabang, Bangkok 10520, Thailand

**Keywords:** chloroplast expression, *Chlamydomonas reinhardtii*, synthetic operon, intercistronic-spacer, biotechnology

## Abstract

Microalgae are a promising platform for generating valuable commercial products, including proteins that may not express well in more traditional cell culture systems. In the model green alga *Chlamydomonas reinhardtii*, transgenic proteins can be expressed from either the nuclear or chloroplast genome. Expression in the chloroplast has several advantages, but technology is not yet well developed for expressing multiple transgenic proteins simultaneously. Here, we developed new synthetic operon vectors to express multiple proteins from a single chloroplast transcription unit. We modified an existing chloroplast expression vector to contain intercistronic elements derived from cyanobacterial and tobacco operons and tested the ability of the resulting operon vectors to express two or three different proteins at a time. All operons containing two of the coding sequences (for *C. reinhardtii FBP1* and *atpB*) expressed the products of those genes, but operons containing the other two coding sequences (*C. reinhardtii FBA1* and the synthetic camelid antibody gene *VHH*) did not. These results expand the repertoire of intercistronic spacers that can function in the *C. reinhardtii* chloroplast, but they also suggest that some coding sequences do not function well in the context of synthetic operons in this alga.

## 1. Introduction

Microalgae have considerable promise as a production platform for proteins and other high-value molecules. Among eukaryotic microalgae, *Chlamydomonas reinhardtii* is the best-studied species and the most amenable to molecular genetic manipulation due to the establishment of efficient transformation methods for all three genomes, expression vectors with inducible and constitutive promoters and multiple selectable marker genes, RNAi, genome editing methods, an indexed insertional mutant library, and other resources [1,2,3]. Already *C. reinhardtii* has been engineered to produce many heterologous proteins and small molecules, including industrial enzymes, therapeutic proteins, antibodies, vaccines, nutraceuticals, and small-molecule metabolites [4,5,6,7,8].

Expression of heterologous proteins in the *Chlamydomonas* chloroplast has a number of advantages compared to expression from the nuclear genome. The chloroplast genome exists in many (~80) copies, transgenes can be targeted to precise locations by homologous recombination, their expression is not silenced by the RNAi machinery as are nuclear transgenes, and fewer proteases reside in the chloroplast than in the cytoplasm [9,10]. However, there are some drawbacks to chloroplast expression, including the fact that there are not yet reliable and efficient tools for gene-stacking in this organelle, which is key for expressing multi-protein complexes or entire enzymatic pathways.

Some efforts have been made to fill this gap. One strategy for expressing multiple transgenes in the chloroplast from a single construct is to integrate multiple transgene cassettes in a single transformation event. Noor-Mohammadi et al. did this to achieve expression of three different reporter genes with individual gene expression cassettes in the chloroplast genome of *C. reinhardtii* [11]. Similarly, Larrea-Alvarez et al. integrated a DNA fragment containing three different gene cassettes (and ultimately a fourth in a subsequent transformation) and achieved expression of all transgenic proteins [12]. This strategy is useful, but the integrated constructs contain three sets of regulatory regions, which increases the size of the construct and also presumably the load on the chloroplast transcription machinery.

An alternative strategy for stacking chloroplast transgenes is polycistronic expression via intercistronic spacer sequences, a method that has been used with success in tobacco and other plants [13,14,15,16,17,18]. To date, there have been two reports relevant to the adaptation of this strategy to *C. reinhardtii*. Su et al. integrated the *apcA/apcB* operon (encoding the α and β subunits of allophycocyanin) from the cyanobacterium *Spirulina maxima* into the *C. reinhardtii* chloroplast and were able to detect expression of both proteins by Western blot [19]. This study served as proof of principle that heterologous proteins could be expressed in *C. reinhardtii* using a transgenic, though technically not synthetic, operon. More recently, Macedo-Osorio et al. used a more systematic approach that employed intercistronic expression elements (IEEs) from several endogenous *C. reinhardtii* chloroplast operons for which there was published evidence of polycistronic transcription [20]. These were the spacer sequences that separate the *psbB-psbT*, *psbN-psbH*, *psaC-petL*, *petL-trnN*, and *tscA-chIN* gene pairs, and they were used in synthetic operons to express *aphA-6* (encodes kanamycin resistance) and *gfp*. Transformants with four of their synthetic operons expressed only the first gene in the operon, but lines that harbored the synthetic operons with the *psbN-psbH* and *tscA-chlN* IEEs produced both aphA-6 protein and GFP. This study represented a breakthrough, as it demonstrated that heterologous proteins could be joined by an IEE/spacer and expressed in the *C. reinhardtii* chloroplast from bi-cistronic messages, and it uncovered two functional intercistronic spacer sequences. However, only two heterologous genes were tested, and the successful intercistronic expression elements were relatively large (569 bp and 650 bp).

Here, we set out to extend these synthetic operon efforts by making and testing vectors with much smaller intercistronic spacers and by testing expression of four different genes in either bi- or tri-cistronic operons. We generated constructs containing either the *Synechococcus elongatus apcA/apcB* spacer or the *Nicotiana tabacum rps19/rpl22* spacer [21] or both to connect up to three of the following genes: *C. reinhardtii FBP1* (nuclear gene cDNA that encodes fructose-1, 6-bisphosphatase, or FBPase); *atpB* (chloroplast gene, encodes β subunit of *C. reinhardtii* chloroplast ATP synthase); *FBA1* (*C. reinhardtii* nuclear gene cDNA that encodes fructose-bisphosphate aldolase); and a synthetic gene that encodes a VHH antibody that targets the bacterial food poisoning agent *Campylobacter jejuni*. In all, we generated and tested nine constructs, six that contain two-gene operons and three that contain three-gene operons, and we integrated them into the chloroplast genome of strains CC-125 or CC-373. CC-125 is wild type, and CC-373 contains a deletion of the *atpB* gene, which is essential for photosynthesis, so the expression of transgenic *atpB* could be easily selected. Phenotypic and Western blot analyses indicated that some of the synthetic operons worked to express the intended transgenic proteins, but not all tested genes were expressed.

## 2. Materials and Methods

### 2.1. C. reinhardtii Strains, Single-Gene Expression Vectors, and Culture Conditions

*C. reinhardtii* strains CC-373 and CC-125 were obtained from the *Chlamydomonas* Resource Center, University of Minnesota. CC-373, carrying the ac-u-c-2-21 mutation, is a light-sensitive, *atpB* deficient strain that has a ~2.5-kb deletion that removes the 3′ part of the *atpB* coding region, its 3′ UTR, and part of one of the chloroplast genome inverted repeats [22]. CC-373 was grown in Tris Acetate Phosphate (TAP) medium (Gorman and Levine, 1965), pH 7.3, in a Percival growth chamber (model#: AR-60L, Percival Scientific, Inc., Perry, IA, USA) at 24 °C in the dark. CC-125 was grown in TAP medium, pH 7.3, in a Percival growth chamber at 24 °C and continuous light (~126 microeinstein per second per square meter, μE m^−2^ s^−1^).

### 2.2. Generation of Plasmid Vectors

Plasmid pFBP1 (previously named pWD-CpFBP1) was described in [23]. Plasmid pFBA1 contains, in place of the *FBP1* sequence in pFBP1, the *C. reinhardtii FBA1* coding sequence (nuclear), gene synthesized (Genscript Biotech Corporation, Piscataway, NJ, USA) to be codon optimized for the *C. reinhardtii* chloroplast and to contain a C-terminal myc tag, flanked by SfoI and MscI restriction sites. Plasmid pVHH contains a similarly codon-optimized fragment encoding a VHH antibody targeting the flagellum of the food poisoning agent *Campylobacter jejuni* [24], but also a 38 amino acid N-terminal Streptavidin-Binding Peptide (SBP) and C-terminal myc tag. Plasmid p-423 (contains the *aadA* gene conferring spectinomycin resistance [25]) was obtained from the Chlamydomonas Resource Center (St. Paul, MN, USA).

**Figure 1:**
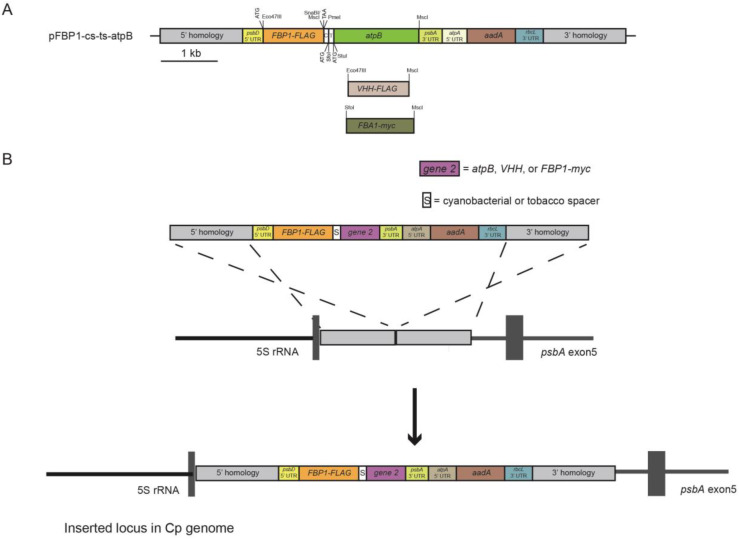
Schematic depiction of synthetic operon construction and integration into chloroplast genome. (**A**) Plasmid pFBP1-cs-ts-atpB was generated by blunt-end ligation of a 1.6-kb gene-synthesized SnaBI-MscI fragment that contains the 55-bp *Synechococcus elongatus apcA/apcB* and 47-bp *Nicotiana tabacam rps*/*rpl* intercistronic spacer sequences upstream of the *C. reinhardtii atpB* coding sequence, into MscI-digested pFBP1. pFBP1-cs-ts-was digested with SfoI and StuI to generate pFBP1-cs-atpB and with PmeI and SfoI to generate pFBP1-ts-atpB. All other synthetic operon constructs analyzed in this study were derived by blunt-end ligation of gene/spacer-gene fragments (including *VHH-FLAG* and *FBA-myc*) into these two synthetic operon vectors to replace or augment existing gene sequences. ATG and TAA indicate locations of start and stop codons, respectively. *aadA*, spectinomycin-resistance coding sequence (from plasmid p-423; [25]. FLAG coding sequence is fused to the 3′ end of *C. reinhardtii FBP1* coding sequence. Boxes labeled 5′ homology and 3′ homology represent DNA fragments identical to sequences in the *C. reinhardtii* chloroplast genome between the 5 S RNA and *psbA* genes. (**B**) Schematic depiction of integration of a synthetic operon into the *C. reinhardtii* chloroplast genome via homologous recombination of 5′ and 3′ homology regions contained by operon vectors and chloroplast genome.

The pFBP1-cs-ts-atpB vector was generated by combining gene-synthesized and existing gene fragments, as described below, and schematic depiction of the strategy for generating this and other synthetic operons is provided in Figure 1A. A 1614-bp DNA fragment was gene synthesized (Genscript) to contain the 55-bp spacer sequence of the *Synechococcus elongatus apc* operon that separates the stop codon of the *apcA* gene and the start codon of the *apcB* gene and the 47-bp *Nicotiana tabacum rps*/*rpl* operon spacer that separates the *rps19* stop codon from the *rpl22* start codon, and the 1470-bp *C. reinhardtii atpB* coding sequence. The cyanobacterial and tobacco spacer sequences were separated by a 9-bp sequence containing a start codon and SfoI restriction site, and that operon cassette was preceded by SnaBI and PmeI restriction sites (surrounding a stop codon) at the 5′ end to permit joining of either operon spacer with downstream *atpB* gene to an upstream gene through blunt-end cloning. The *atpB* coding region was synthesized to contain a StuI restriction site immediately following its start codon and an MscI restriction site immediately following its final codon. The gene-synthesis fragment was digested with SnaBI and MscI to liberate a 1608-bp blunt-ended fragment that was ligated into MscI-digested pFBP1 to generate p-FBP1-cs-ts-atpB. p-FBP1-cs-ts-atpB was used to generate the nine chloroplast expression vectors tested in this study, which contain different combinations of the *FBP1*, *atpB*, *FBA1*, and *VHH* genes connected by cyanobacterial and/or tobacco chloroplast operon spacers as described below.

pFBP1-cs-atpB and pFBP1-ts-atpB, which contain the *FBP1* and *atpB* genes connected by the cyanobacterial spacer or the tobacco spacer, respectively, were generated as follows. pFBP1-cs-ts-atpB was digested with restriction enzymes SfoI and StuI (Thermo Fisher Scientific, Waltham, MA, USA) and then religated to produce pFBP1-cs-atpB, and it was digested with PmeI and SfoI and then religated to produce pFBP1-ts-atpB.

pFBP1-cs-VHH and pFBP1-ts-VHH, which contain the *FBP1* and *VHH* genes connected by the cyanobacterial spacer or the tobacco spacer, respectively, were generated as follows. First, pFBP1-cs-atpB was digested with SfoI and MscI to remove the fragment containing the *atpB* gene and cyanobacterial spacer, and the remaining 9.355-kb vector fragment from this digest was ligated with the 1005-bp coding sequence fragment of the *VHH* gene derived from a Eco47III and MscI digest of vector pVHH; the completed expression vector was named pFBP1-cs-VHH. pFBP1-ts-VHH was generated by ligating the Eco47III-MscI *VHH* fragment into the vector fragment produced by StuI and MscI digestion of pFBP1-ts-atpB.

pFBP1-cs-FBA and pFBP1-ts-FBA, which contain *FBP1* and *FBA1* genes connected by the cyanobacterial spacer or the tobacco spacer, respectively, were generated as follows. pFBP1-cs-FBA was produced by ligating the 1170-bp coding sequence fragment of *FBA1* that was liberated by digesting pFBA1 with SfoI and MscI into the vector fragment derived from a digest of pFBP1-cs-atpB with StuI and MscI. Similarly, pFBP1-ts-FBA was generated by ligating the 1170-bp SfoI-MscI *FBA1* fragment into the vector fragment derived from a StuI + MscI digest of pFBP1-ts-atpB.

Expression vectors pFBP1-cs-FBA-cs-atpB, pFBP1-cs-FBA-ts-atpB, and pFBP1-ts-FBA-ts-atpB were generated as follows. The 1542-bp fragment containing the cyanobacterial spacer and *atpB* gene coding sequence was liberated by digesting pFBP1-cs-atpB with PmeI and MscI, then was ligated into the vector fragment derived from an MscI digest of pFBP1-cs-FBA to generate pFBP1-cs-FBA-cs-atpB. Similarly, the 1532-bp SfoI-MscI fragment from pFBP1-ts-atpB was ligated into MscI-digested pFBP1-cs-FBA to produce pFBP1-cs-FBA-ts-atpB. Finally, the 1532-bp SfoI-MscI fragment from p-FBP1-cs-ts-atpB containing the tobacco operon spacer and *atpB* coding sequence was ligated into MscI-digested pFBP1-ts-FBA, to generate pFBP1-ts-FBA-ts-atpB. All final constructs were verified by diagnostic restriction digestions and sequencing (primers displayed in Table 1) to verify expected fragment sizes, to determine that no unintended sequence changes had occurred during cloning steps, and to verify that insert fragments were all in correct orientation.

### 2.3. C. reinhardtii Chloroplast Transformation

Chloroplast transformation was performed using the biolistic particle bombardment method as described previously [23]. Strain CC-373 cells were cultured in 25 mL TAP medium with dim light for seven days to a density of 3 × 10^6^ cells/mL. The culture was then centrifuged at 3000× *g* for 5 min, and the pellet was resuspended in TAP medium to a concentration of 30 × 10^6^ cells/mL. In total, 330 µL of the resuspended cells was spread onto a TAP-agar plate supplemented with 100 µg/mL spectinomycin and allowed to dry, uncovered, in a dark sterile hood for 10 min.

Five macrocarriers (Bio-Rad Laboratories, Inc., Hercules, CA, USA) were washed in absolute ethanol and assembled into macrocarrier holders, then placed in a desiccator for 2–3 h to dry. DNA coating of gold particle microcarriers (550 µM diameter; SeaShell Technology, LLC, La Jolla, CA, USA) was as follows: 50 µL of binding buffer was mixed with 10 µL of plasmid, 1 µg/µL, in a sterile 1.5 mL microcentrifuge tube; 60 µL of gold carriers (50 mg/mL) and 100 µL of precipitation buffer were added to the mixture. The mixture was briefly centrifuged for 10 s at 12,000× *g*, and the supernatant was removed. The pellet was washed with 500 µL cold absolute ethanol, and the supernatant was removed. The pellet was resuspended in 50 µL of absolute ethanol, and 10 µL of DNA-coated microcarriers were loaded onto the inner area of a macrocarrier. The macrocarrier was dried for 10 min in a desiccator.

The plasmid DNA coated with gold particles was bombarded into *C. reinhardtii* cells via PDS-100/He gene gun (Bio-Rad Laboratories, Inc., USA) using helium gas and a 1350-psi rupture disk. After bombardment, the plates were incubated in a Percival growth chamber with dim light for approximately 3 weeks. Transformant colonies appeared after ~16 days.

For wild-type strain CC-125, the preparation of cells and plasmid DNA coating of microcarriers were carried out as described above. However, CC-125 cells were cultured under continuous light for four days prior to bombardment, after which the plates were incubated in dim light overnight, then placed under continuous light for two weeks.

### 2.4. Genomic DNA Extraction and Colony PCR

Genomic DNA was extracted using the Chelex method, as described below; 1–5 × 10^6^ *C. reinhardtii* cells from transformant and recipient strains were added to 60 µL of Chelex 100 solution buffer (0.1 M NaOH + 5% Chelex-100 (Bio-Rad) and the mixture vortexed to resuspend the resin, in a microcentrifuge tube. The samples were boiled at 65 °C for 15 min and centrifuged at 11,000× *g* for 1 min. In total, 20 µL of the supernatant was transferred into 30 µL of 50 mM TrisCl, pH 8, and stored at −20 °C. The cell lysate was used as a template DNA for PCR. For some samples, the colony PCR method was used, described in [26]. Briefly, 1 µL of genomic DNA extracts from transformants, and recipient strains (prepared as described above) were inoculated into 19 µL of DreamTaq master mix (Thermo Fisher Scientific, Inc.), primer sets, and dH_2_O. Gene-specific forward and reverse primers (Invitrogen) are listed in Table 1. All PCR were performed using a T100^TM^ Thermal Cycler (Bio-Rad) for 29 cycles under the following thermal cycle conditions: 98 °C for 30 s, 98 °C for 30 s, 54 °C for 30 s, and 72 °C for 2 min, with a final extension at 72 °C for 10 min. DNAs from transformant cells were amplified with primer sets P1-P5 to screen for the presence of the genes *FBP1*, *atpB*, *VHH*, and *FBA1* in integrated transgene cassettes, respectively.

**Table 1:** List of oligonucleotide primers for sequencing and colony PCR.

Name	Sequence (5′-3′)	Purpose
Syn-op1	CCAGAAAAAGTTCATCAACGTGTACCTTTATT	Sequencing
Syn-op2	TACACGTTGATGAACTTTTTCTGG	Sequencing
Syn-op3	AGCTTCAACAGGTTCATTTAAAGG	Sequencing
P1 (*FBP1*)	F: GCACAAAGCAGTTCTAGTCC R: ACCATCAATTGTACCTTTCATCTC	Genomic PCR
P2 (*atpB*)	F: AACATTTTCCGTTTCGTACAAGCT R: GTCCTGCCAACTGCCTATG	Genomic PCR
P3 (*VHH*)	F: AGGTAGTTATCAATATTGGGGTCA R: GTCCTGCCAACTGCCTATG	Genomic PCR
P4 (*FBA1*)	F: TACATTAGGTCCAGGTGATTATTC R: AGGAGCTGTACGGTGAATTGGTAA	Genomic PCR
P5 (*FBA1*/*atpB*)	F: TACATTAGGTCCAGGTGATTATTC R: AGGAGCTGTACGGTGAATTGGTAA	Genomic PCR

### 2.5. Phenotypic Screening for atpB Transformants

A total of 16 days after bombardment, cells from surviving spectinomycin-resistant colonies were streaked onto acetate-free Tris Phosphate (TP) medium plates (medium titrated with HCl to pH 7.0) supplemented with 100 µg/mL spectinomycin and grown in a Percival growth chamber under continuous light and at 24 °C for 5–7 days to assay for photoautotrophic growth.

### 2.6. Western Blot Analysis

Western blots to assay accumulation of transgenic protein were performed as described previously [23] with minor modifications. Cells of transformants were cultured in 25 mL TAP medium (supplemented with 100 µg/mL spectinomycin) to mid-log phase, pelleted by centrifugation at 3000× *g* for 5 min, mixed with 200 µL lysis buffer (4% SDS, 100 mM Tris pH 6.8, 10% glycerol, and 100 mM DTT) and boiled for 5 min. Protein extracts from the cells were loaded onto 12% SDS-PAGE gels and electrophoresed at 100 V for 85–100 min. The proteins were transferred to a nitrocellulose membrane (Amersham-Protran; GE Healthcare, Silver Spring, MD, USA) in a Mini Protean Tetra Cell (Bio-Rad) set to 21 V and 50 milliamps. The following day, the membrane was blocked with 5% milk (10% Carnation non-fat) for one hour. To detect FBP1 and VHH proteins, the membrane was probed with a primary anti-FLAG mouse monoclonal antibody (Sigma-Aldrich, Inc., St. Louis, MO, USA) diluted 1:2000 in 2 mL of 1X PBST (0.01% Tween in phosphate-buffered saline) with 0.06 g Bovine Serum Albumin (BSA) (Sigma-Aldrich) for 1 h. The membrane was then probed with a secondary goat anti-mouse IgG antibody-HRP conjugate (diluted 1:40,000 in 40 mL of 5% milk solution) for 30 min. To detect atpB protein, the membrane was reacted with a primary rabbit polyclonal anti-atpB-antibody (Agrisera, Vännäs, Sweden) diluted 1:2000 in 2 mL of 1X PBST with 0.06 g BSA for one hour, then probed with a secondary goat anti-rabbit IgG antibody diluted 1:40,000 in 40 mL 5% milk solution for 30 min. To detect FBA1 protein, membranes were probed with a primary anti-myc mouse monoclonal antibody (Active Motif, Carlsbad, CA, USA) diluted 1:2000 in 2 mL of 1X PBST with 0.06 g BSA for one hour. After that, the membrane was probed with a secondary goat anti-mouse IgG-HRP conjugate antibody diluted at 1:40,000 in 40 mL of 5% milk solution for 30 min. The membrane was washed 5 times with 1X PBST, developed with Super Signal West Femto Maximum Sensitivity Substrate (Thermo Fisher Scientific) for 5 min, and exposed to X-ray film.

## 3. Results

### 3.1. Cyanobacterial and Tobacco Operon Spacer Sequences can Function in Synthetic C. reinhardtii Chloroplast Operons

**Figure 2:**
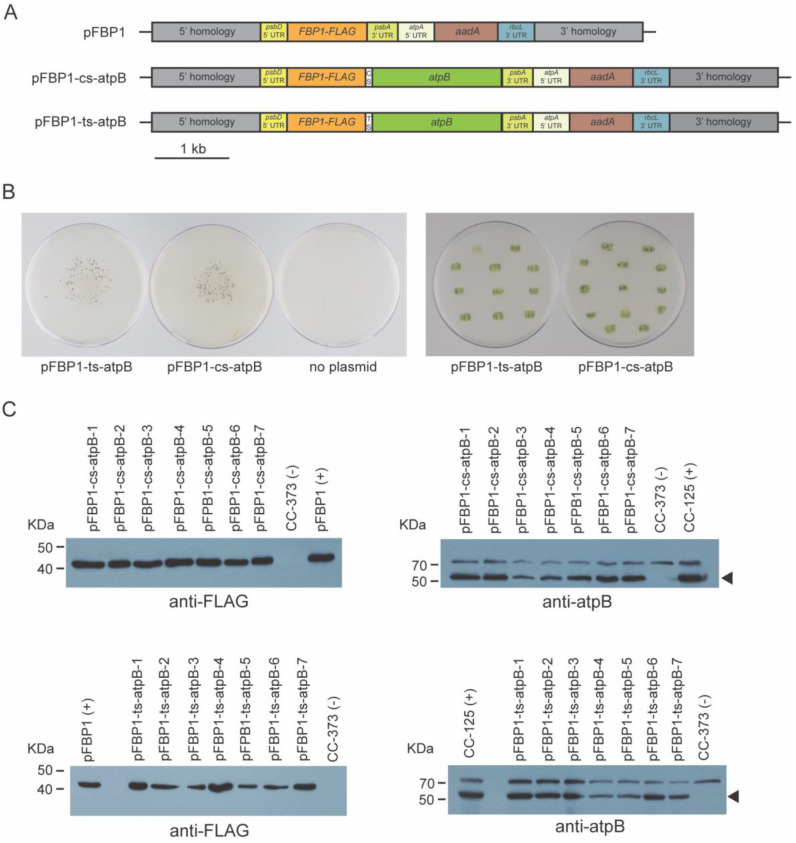
Mutant rescue and Western blot analysis of synthetic *FBP1-atpB* operon transformants. (**A**) Schematic depiction of synthetic operon vectors pFBP1-cs-atpB and pFBP1-ts-atpB, and control vector pFBP1. cs, cyanobacterial spacer; ts, tobacco spacer. (**B**) Transformation plates showing colonies surviving spectinomycin selection (left panel), and restreak plates showing survival of transformants on tris phosphate medium in light (right panel); Nearly all tested transformants survived phototrophic selection. No survivors were obtained for control bombardment that did not include a chloroplast transformation vector (third plate, left panel). (**C**) Western blot analysis of FBPase and atpB proteins in pFBP1-cs-atpB and pFBP1-ts-atpB transformants. Proteins from seven pFBP1-cs-atpB transformants (top panels) and seven pFBP1-ts-atpB transformants (bottom panels) were electrophoresed, transferred to nitrocellulose membrane, and reacted with anti-FLAG antibody (left two panels), and anti-atpB antisera (right two panels). Extract from recipient strain CC-373 served as negative control for all blots, extract from wild-type strain CC-125 (right panels) or from CC-125 harboring the insert from pFBP1 (left panels) served as positive controls. Arrowheads in right panels indicate position of atpB protein to distinguish it from a non-specific > 70 kDa protein detected by the antisera.

To test the idea that short operon spacers derived from other genomes might be used in vectors for simultaneous expression of multiple proteins in the chloroplast genome of *C. reinhardtii*, we made two versions of chloroplast expression vectors containing the nuclear *C. reinhardtii* gene *FBP1* and the chloroplastic *C. reinhardtii* gene *atpB* within a synthetic operon. pFBP1-cs-atpB contains a cyanobacterial *apcA/apcB* operon spacer, which connects *FBP1-FLAG* with *atpB* (Figure 2A). pFBP1-ts-atpB contains a tobacco chloroplast *rps19/rpl22* operon spacer positioned between the same two coding sequences. In each vector, stop codons follow the *FBP1* and *atpB* coding sequences, and the *FBP1*-spacer-*atpB* fragment is flanked by upstream *psbD* 5′ regulatory/UTR sequence and downstream *psbA* 3′ UTR. The vector also contains a spectinomycin resistance gene cassette (Figure 2A). The synthetic operon sequence is sandwiched between 5′ and 3′ homology regions that target integration to the chloroplast genome, as previously described (Figure 1B [23]). Our strategy was to select spectinomycin transformants, then test these for rescue of the photosynthesis-related growth defects of CC-373, then examine rescued transformants for FLAG-tagged FBPase and atpB protein accumulation.

We obtained several dozen spectinomycin-resistant transformants with each vector (Figure 2B). To determine whether the transformants contained at least pieces of the *FBP1* and *atpB* gene cassettes, we performed colony PCR with genomic DNA of seven transformant strains for each construct. We could amplify a region between the 3′ end of 5′ *psbD* regulatory sequences and the 5′ end of the *FBP1* coding sequence and a region between the 3′ end of the *atpB* coding sequence and the 5′ end of 3′ *psbA* regulatory sequences from all seven transformants for each construct, confirmed that the *FBP1* CDS and *atpB* CDS cassettes were successfully transformed into CC-373 (Figure S1).

We next tested the transgenic strains for rescue of the *atpB* deletion, which renders the parent strain (CC-373) light sensitive and unable to photosynthesize. Nearly all transformants that we analyzed survived in light without acetate (Figure 2B), demonstrating that *atpB* gene function was restored. Consistent with this result, Western blot analysis using an anti-atpB antibody demonstrated that all tested transformants receiving either pFBP1-cs-atpB or pFBP1-ts-atpB expressed a protein at the size expected for atpB (~53 kDa; Figure 2C). Furthermore, all transformants receiving either synthetic operon construct contained a FLAG-positive protein that was the size expected for FBP1-FLAG (~41 kDa; Figure 2C). Neither protein was present in recipient strain CC-373. These results demonstrate that two open reading frames can be expressed in the *C. reinhardtii* chloroplast from a single transcription unit when they are separated by the cyanobacterial or tobacco operon spacers.

### 3.2. Synthetic Operons with Cyanobacterial and Tobacco Spacers Do Not Express All Tested Genes

**Figure 3:**
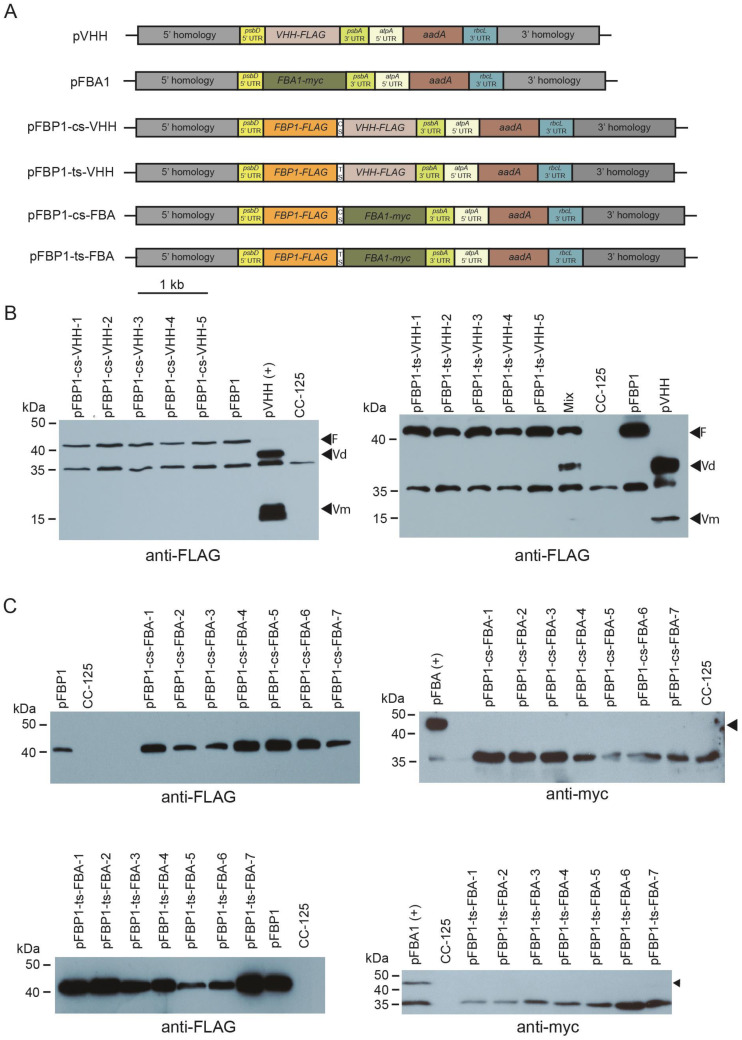
Western blot analysis of synthetic *FBP1-VHH* and *FBP1-FBA1* operon transformants. (**A**) Schematic depiction of synthetic operon vectors pFBP1-cs-VHH, pFBP1-ts-VHH, pFBP1-cs-FBA, and pFBP1-ts-FBA, and control vectors pVHH and pFBA. cs, cyanobacterial spacer; ts, tobacco spacer. (**B**) Western blot analysis of FBP1 and VHH proteins in pFBP1-cs-VHH and pFBP1-ts-VHH transformants. Proteins from five pFBP1-cs-VHH transformants (left panel) and five pFBP1-ts-atpB transformants (right panel) were electrophoresed, transferred to nitrocellulose membrane, and reacted with anti-FLAG antibody. Extract from recipient strain CC-125 served as negative control, and extract from CC-125 harboring the insert from pFBP1 or pVHH served as positive controls. Arrowheads indicate position of FLAG-tagged FBPase protein (F) and of either the dimer (Vd) or monomer (Vm) versions of VHH protein. A non-specific protein of ~35 kDa is also detected by the anti-FLAG antibody in strain CC-125. (**C**) Western blot analysis of FBPase-FLAG and FBA1-myc proteins in pFBP1-cs-FBA and pFBP1-ts-FBA transformants. Proteins from seven pFBP1-cs-FBA (top panels) and seven pFBP1-ts-FBA (bottom panels) transformants were electrophoresed, transferred to nitrocellulose membranes, and reacted with anti-FLAG antibody (left panels) or anti-myc antibody (right panels) to detect transgenic FBP-FLAG and FBA1-myc proteins, respectively. Transgenic FBPase was present in all 14 transformants but not in the recipient strain CC-125. None of the transformants accumulated FBA1 protein. Arrowheads in right panels indicate position of FBA1-myc protein. A non-specific band was detected at ~35 kDa for the anti-myc antibody blots.

To determine whether the cyanobacterial *apcA/apcB* and tobacco chloroplast *rps19/rpl22* operon spacers might function with other genes in the second position, we generated new synthetic operons that are identical to the vectors described above, except that the *atpB* coding sequence was replaced either by a FLAG-tagged version of a gene (*VHH-FLAG*) that encodes a single-chain antibody against the food-poisoning agent *Campylobacter jejuni* or by a myc-tagged version of the *C. reinhardtii FBA1* coding sequence (*FBA1-myc*). We chose these genes because we could express them in the *C. reinhardtii* chloroplast using standard single-gene chloroplast expression vectors pFBA and pVHH that express easily detectable levels of VHH-FLAG and FBA-myc (Figure 3).

Bombardment of pFBP1-cs-VHH and pFBP1-ts-VHH into wild-type strain CC-125 yielded ~50 spectinomycin-resistant *C. reinhardtii* colonies for each plasmid. As for the pFBP1-cs-atpB and pFBP1-ts-atpB transformants, colony PCR analysis showed that the two transgenic ORFs were present in all transformants analyzed (Figure S2).

We next performed Western blot analysis to determine if *FBP1-FLAG*, *FBA-myc*, and *VHH-FLAG* were expressed from the synthetic operons. Protein extracts were prepared from the recipient line, from lines containing pFBP and pVHH, and from spectinomycin-resistant lines that were shown by genomic PCR to contain the transgenes. All tested transformants for both synthetic operons accumulated protein of the size expected for FBPase, while neither VHH-FLAG nor FBA1-myc was present in CC-125, the negative control. However, no tested transformants that contained the synthetic operon constructs pFBP1-cs-VHH or pFBP1-ts-VHH accumulated VHH protein, either the ~37 kDa dimeric or the ~17 kDa monomeric forms, and likewise, no transformants containing pFBP1-cs-FBA or pFBP1-ts-FBA had detectable levels of FBA1 protein (expected ~43 kDa) (Figure 3). In the case of the pFBP1-ts-VHH and pFBP1-ts-VHH transformants, we considered the possibility that FBP1-FLAG protein in the transformant extracts might somehow titrate out the anti-FLAG antibody and prevent it from binding VHH, but mixing the pFBP1-ts-VHH extract with the pVHH positive control extract did not prevent the antibody from detecting VHH-FLAG (Figure 3B, right panel, lane 6). These results indicate that unlike the situation for pFBP1-cs-atpB and pFBP1-ts-atpB, the second gene in these synthetic operons was not expressed, suggesting that not all genes can express well from the second position in these two-gene synthetic operons.

### 3.3. Sandwiching FBA1 Coding Sequence between That for FBP1 and atpB in a Three-Gene Operon Does Not Improve Its Expression

**Figure 4:**
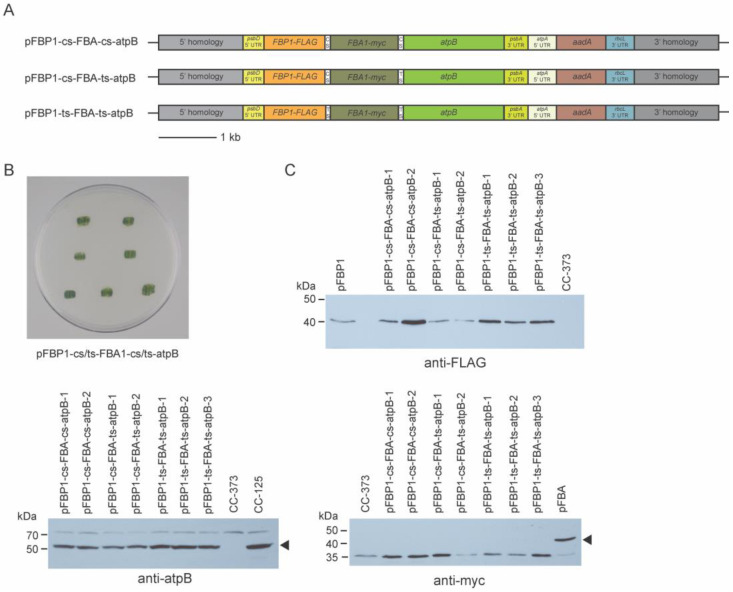
Mutant rescue and Western blot analysis of 3-gene synthetic operon transformants. (**A**) Schematic diagrams of synthetic operon vectors pFBP1-cs-FBA-cs-atpB, pFBP1-cs-FBA-ts-atpB, and pFBP1-ts-FBA-ts-atpB. cs, cyanobacterial spacer; ts, tobacco spacer. (**B**) All spectinomycin-resistant survivors from bombardment of *atpB* mutant strain CC-373 with three-gene operon constructs were restruck on tris phosphate medium plates and grown in the light; all transformants survived phototrophic selection, demonstrating rescue of the *atpB* mutant phenotype. Top two patches, pFBP1-cs-FBP1-cs-atpB; middle two patches, pFBP1-cs-FBP1-ts-atpB; bottom three patches, pFBP1-ts-FBP1-ts-atpB (**C**) Western blot analysis of 3-gene synthetic operon transformants. Proteins from synthetic operon transformants, negative control recipient strain CC-373, and positive controls CC-125 or CC-125 transformed with either pFBP1 or pFBA were electrophoresed and transferred to a nitrocellulose membrane. Blots were reacted with anti-FLAG antibody to detect FBPase-FLAG protein (top right panel), with anti-myc antibody to detect FBA1-myc (bottom right panel), and with anti-atpB antibody (bottom left panel). Arrowheads indicate positions of FBA1-myc and atpB to distinguish from proteins that react nonspecifically with the anti-myc and anti-atpB antisera, respectively.

To determine whether it might be possible to force expression of *FBA1* by positioning it between two ORFs (*FBP1* and *atpB*) that presumably would be expressed, we generated three new synthetic operon constructs that contain *FBP1-FLAG* in the first position, *FBA1-myc* in second, and *atpB* in the third, and with three different combinations of cyanobacterial and tobacco spacers (Figure 4A).

These three synthetic operon constructs were transformed separately by biolistic particle bombardment into the chloroplast genome of recipient strain CC-373. A small number (from two to six) of spectinomycin-resistant colonies were obtained with these constructs, and genomic PCR analysis on a subset of these determined that they had received all three genes (Figure S3). All of these transformants survived in light without acetate (Figure 4B), demonstrating that all transformants were capable of photosynthetic growth and must have expressed the *atpB* transgene, indicating robust expression from the third gene in the three-gene operon.

To determine FBPase-FLAG, FBA1-myc, and atpB accumulation in the transformants, protein extracts from each of two transformants receiving pFBP1-cs-FBA-cs-atpB and pFBP1-cs-FBA-ts-atpB and from three transformants for pFBP1-ts-FBA-ts-atpB were subjected to Western blot analysis. All transformants receiving the synthetic operons expressed proteins with expected sizes for FBPase-FLAG and for atpB but not for FBA1-myc (Figure 4B). These results indicate that the synthetic operons containing three genes worked to express protein from the first (*FBP1*) and third (*atpB*) ORFs, but they failed to express protein from the second ORF (*FBA1*), meaning that expression of the first and third ORFs was not sufficient to ensure expression of the second one.

## 4. Discussion

As part of a prior investigation, we reported overexpression of *C. reinhardtii* FBPase in the chloroplast using a newly generated expression vector [23]. Here, with the goal of improving algal chloroplast protein expression technology, we used that vector as a starting point to generate synthetic operons that contain previously untested intercistronic spacer sequences (spacers), one derived from the cyanobacterial species *S. elongatus* and the other from the tobacco chloroplast genome. These spacers (*apcA/apcB* and *rps19/rpl22*), at 56 and 47 bp, respectively, are >10 times smaller than the *C. reinhardtii* intergenic expression elements shown recently to work in the first (and thus far only other) synthetic operons reported to function in *C. reinhardtii* [20]. Synthetic operons in which either the cyanobacterial or the tobacco spacer separated the *FBP1* and *atpB* genes worked to express both encoded proteins, showing that these short spacers from disparate, heterologous species can function well in *C. reinhardtii* chloroplast operons. However, all operons containing *FBP1* and either of two other genes, *C. reinhardtii FBA1* or a gene that encodes a synthetic camelid heavy-chain variable domain (VHH) antibody, expressed only the *FBP1* gene product and not the *FBA1* or *VHH* products. This result was surprising given that both FBA1 and VHH protein products were produced at easily detectable levels when either was expressed in a single-gene construct, and it raises the possibility that sequence-specific elements within coding regions of genes, or more generally, the overall nature of that coding sequence, might have strong effects on protein expression from synthetic operons in *C. reinhardtii*. It also raises the question of how commonplace it will be for genes in the second position to express well with the *apcA/apcB* and *rps19/rpl22* spacers. Future efforts should include testing additional genes in that position, including *FBP1*.

We chose to use the *apcA/apcB* and *rps19/rpl22* spacers in our synthetic operons because their small size (they are among the shortest intercistronic spacers we could find among published prokaryotic or chloroplast genomes) helped to make the synthetic operons compact and easy to manipulate. Indeed, these spacers are at least 500 bp smaller than the spacers/IEEs used in the previously reported *C. reinhardtii* synthetic operons [20]. Small spacer size should be especially advantageous for efforts to stack three or more cistrons into the same operon. For instance, it might be difficult or impossible to integrate inserts above a certain size into the chloroplast genome. Interestingly these two spacers are not similar at all to each other at the primary sequence level, except that both are fairly A/T rich (64.3–72%), suggesting that the requirements for spacer function in a synthetic operon might be relatively flexible. However, there must be some requirements, at least for some synthetic operons. Macedo-Osorio et al. found that only two of the six endogenous IEEs/intercistronic spacers they tested functioned in their *C. reinhardtii* synthetic operon constructs [20], and Lu et al. found that processing of a synthetic operon primary transcript in tobacco was defective when they used a spacer derived from an intergenic region of the chloroplast instead of an intercistronic spacer from the *psbB* operon [17].

A possible disadvantage of small spacers is that they might not contain regulatory sequences required for the stability of processed versions of some synthetic operon transcripts. At least some polycistronic chloroplast mRNAs in higher plants are initially processed, presumably by endonucleases that cleave A/U-rich regions within the intercistronic spacer, after which those post-cleavage transcripts are believed to be stabilized by pentatricopeptide repeat (PPR) or tetratricopeptide repeat (TPR) proteins that bind within an intercistronic RNA sequence, blocking exonuclease-mediated degradation [27,28,29]. Many *C. reinhardtii* chloroplast mRNAs are polycistronic, and some of these are also processed into monocistronic units [20,29,30,31,32], though little is known about how these specific processing events are regulated in this species.

Another possible disadvantage of small spacers is that they might not permit the required secondary structure for the unmasking of the ribosome binding site (RBS) in the second cistron of polycistronic mRNAs. In our experiments, the small spacers did work for *atpB*, possibly because the sequence of this gene near the RBS is such that the RBS is not sequestered within a double-stranded RNA region, but the secondary structure situation might be very different for the *VHH* and *FBA* genes. Conceivably even larger spacers, such as the ones used in [20], might contain sequences that lead to masking of the RBS for some downstream genes, explaining why some of the synthetic operons tested in that study did not work. A follow-up analysis of accumulated transcripts in transformants that did not express the second gene in the synthetic operon could shed some light on this question.

In general, the coding sequence of the downstream gene in a synthetic operon might greatly influence the stability of the processed transcripts. It seems possible that the *FBA1* and *VHH* coding sequences lack features required for post-processing stability, which could explain why those genes could be expressed from single-gene vectors but did not work in our synthetic operons, even when they were sandwiched between two cistrons that were translated (Figure 4). Consistent with this idea, it has been shown that sequences within the coding region of a *C. reinhardtii* chloroplast gene can have dramatic effects on transcript accumulation [33].

Given these considerations, in the future, it might be helpful to use computational methods to predict (and then subsequently alter) transcript structural features when designing synthetic operons. Secondary structure predictions were previously used to guide the modification of a single-gene *C. reinhardtii* chloroplast expression vector. In this case, mutating the 5′ UTR to alter predicted stem structures had profound effects on translation [34]. Another strategy that has promise is to engineer into the spacer sequence the binding site for a known PPR or TPR protein to stabilize the processed transcripts that might arise from the synthetic operon. As a variation of that strategy, PPR or TPR proteins could be engineered to bind and stabilize existing spacer sequences. To that end, a combinatorial amino acid code has been worked out for the helical repeat motifs of some plant PRR proteins, making it possible to design these proteins to have specificity for a wide variety of RNA sequences [35,36]. In conclusion, our findings represent an advance in *C. reinhardtii* synthetic operon technology, but they also serve as a reminder that gaps in our understanding of chloroplast RNA processing and translation in this species might need to be filled to realize the potential of this method. Further investigation of how intercistronic spacer sequences function, both those native to *C. reinhardtii* and those from other species, will likely be necessary to fully harness this approach.

## Data Availability

No new data were created that were not reported in this manuscript.

## Supplementary Materials

The following supporting information can be downloaded at: https://www.mdpi.com/article/10.3390/genes14020368/s1, Figure S1: Genomic PCR analysis of pFBP1-cs-atpB and pFBP1-ts-atpB transformants.; Figure S2: Genomic PCR analysis of pFBP1-cs-atpB and pFBP1-ts-atpB transformants.; Figure S3: Genomic PCR analysis of pFBP1-cs-FBA-cs-atpB, pFBP1-cs-FBA-ts-atpB, and pFBP1-ts-FBA-ts-atpB transformants.
