# Supplementary material for: New Synthetic Operon Vectors for Expressing Multiple Proteins in the Chlamydomonas reinhardtii Chloroplast
Source: Genes (Basel). 2023 Jan 31;14(2):368. doi: 10.3390/genes14020368 (PMC9956277; doi:10.3390/genes14020368)
Supplement: Supplementary file 1 [file genes-14-00368-s001.zip › genes-2168379-supplementary.pdf]

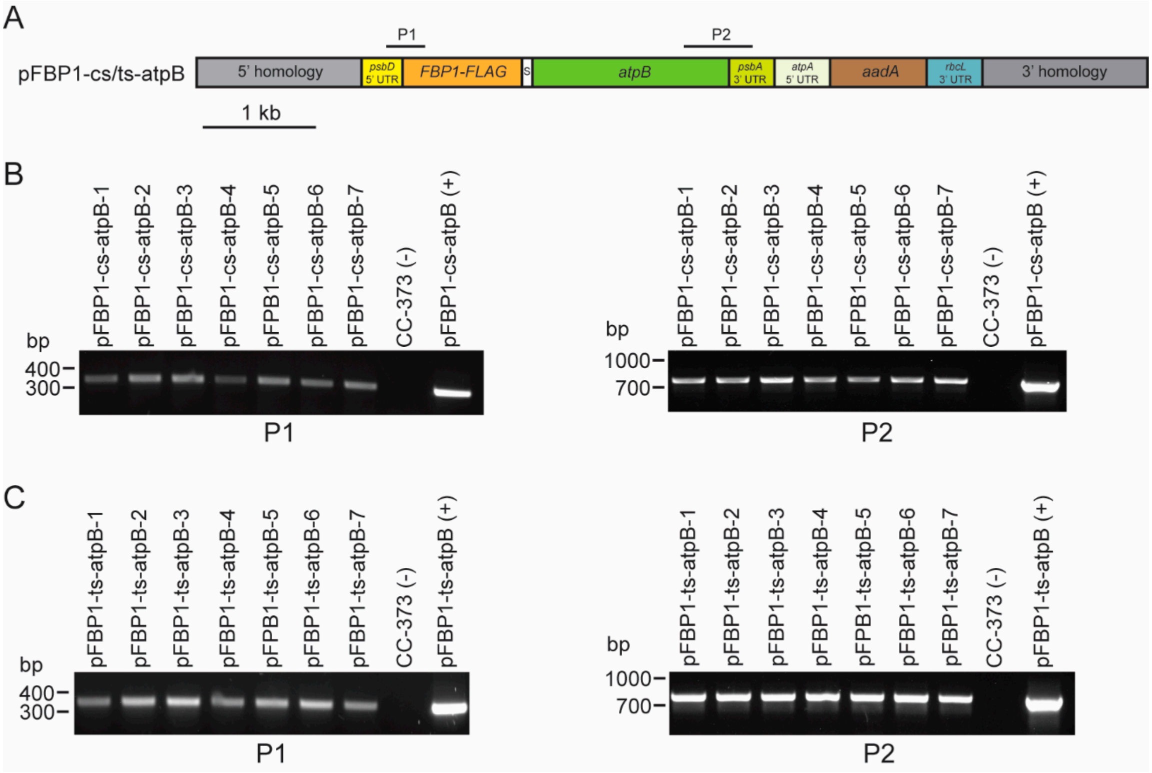

Figure S1. Genomic PCR analysis of pFBP1-cs-atpB and pFBP1-ts-atpB transformants. (A) Schematic diagram depicting the synthetic operon constructs that were bombarded into strain CC-373, showing regions of the chimeric *FBP1* and *atpB* genes amplified by primer sets P1 and P2. White box labeled s, spacer. (B) Genomic PCR analysis of pFBP1-cs-atpB transformants using primer set P1, and (C) genomic PCR analysis of pFBP1-ts-atpB transformants using primer set P2. Genomic DNA was extracted from transformants and from recipient strain CC-373, which served as a negative control. The transformed plasmids were used as positive controls for each set of PCR.

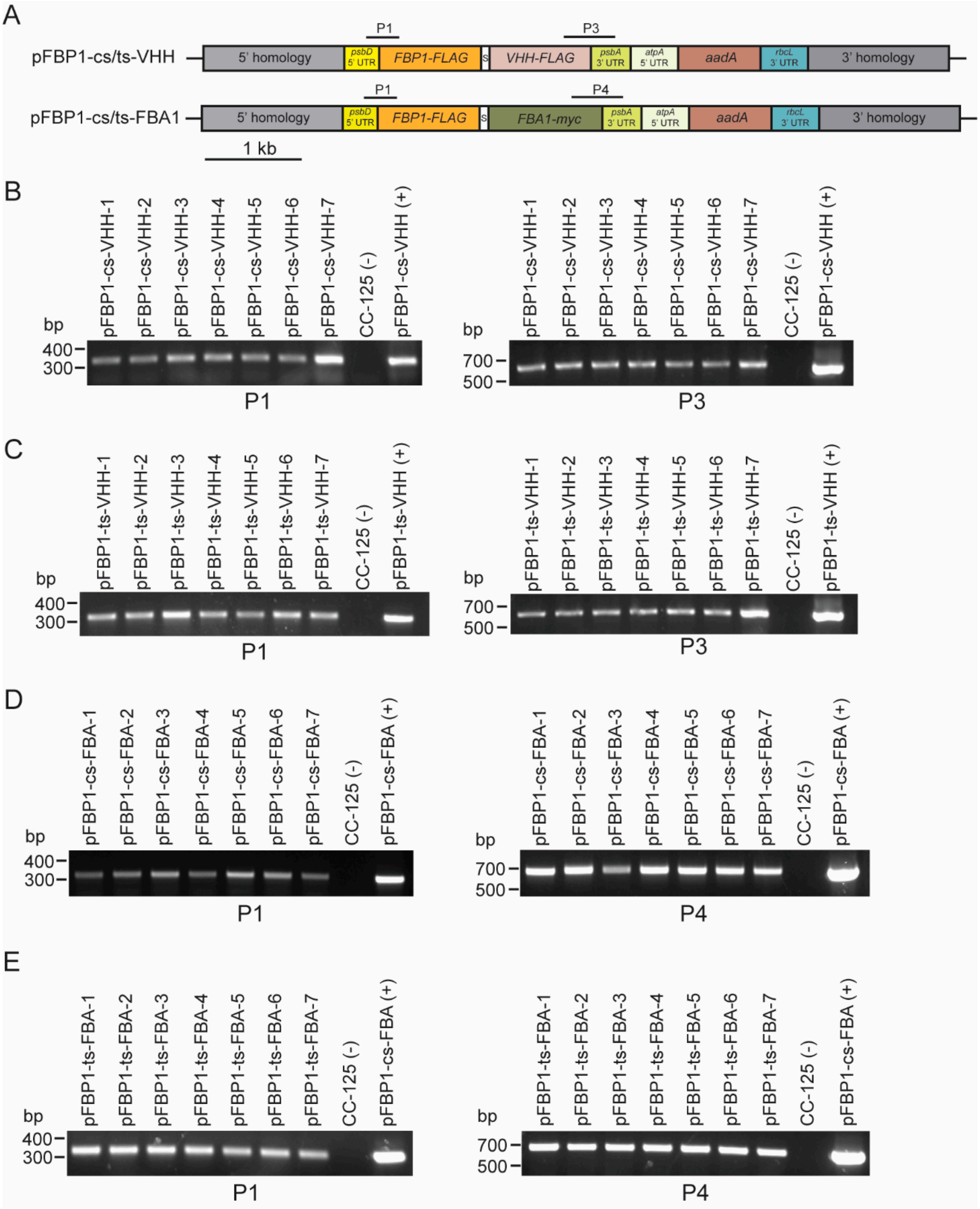

Figure S2. Genomic PCR analysis of pFBP1-cs-VHH, pFBP1-ts-VHH, pFBP1-cs-FBA, and pFBP1-ts-FBA transformants. (A) Schematic diagrams depicting the synthetic operon constructs (containing *FBP1-FLAG* and either *VHH-FLAG* or *FBA1-myc* coding sequences) that were bombarded into cells of strain CC-125, showing regions of the chimeric *FBP1*, *VHH*, and *FBA1* genes amplified by primer sets P1, P3, and P4. White box labeled s, spacer. (B) and (C), genomic PCR analysis of pFBP1-cs-VHH and pFBP1-ts-VHH transformants, respectively using primer sets P1 and P3 to detect chimeric *FBP1* (left panels) and *VHH* (right panels). (D) and (E), genomic PCR analysis of pFBP1-cs-FBA and pFBP1-ts-FBA transformants, respectively, using primer sets P1 and P4 to amplify a region of chimeric *FBP1* (left panels) and a region of chimeric *FBA1* (right panels). Genomic DNA was extracted from transformants and from recipient strain CC-125, which served as negative control. The transformed plasmids were used as positive controls for each set of PCR.

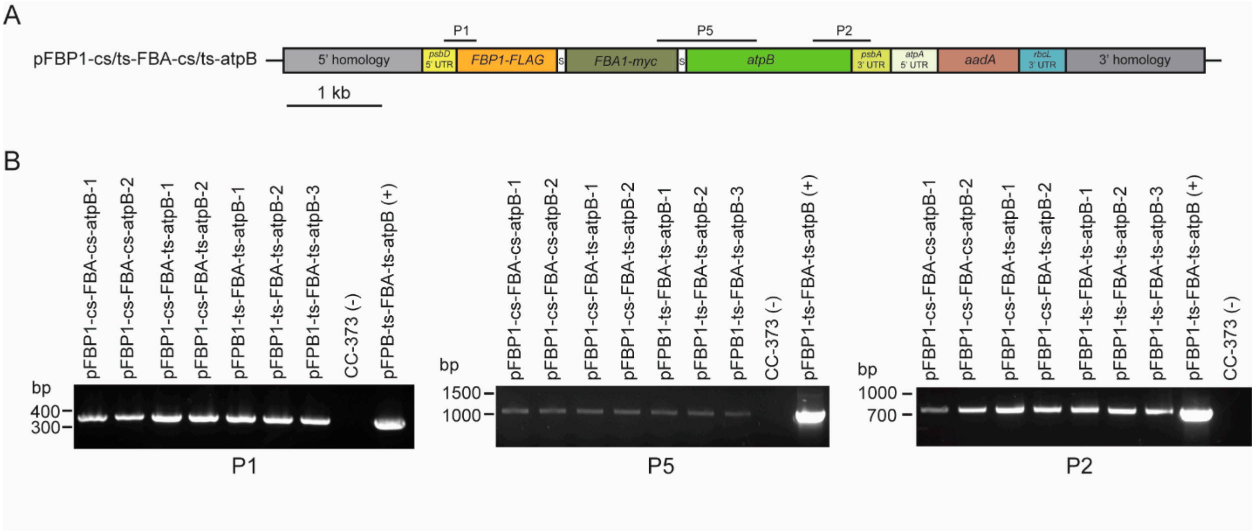

Figure S3. Genomic PCR analysis of pFBP1-cs-FBA-cs-atpB, pFBP1-cs-FBA-ts-atpB, and pFBP1-ts-FBA-ts-atpB transformants. Schematic diagrams depicting the 3-gene synthetic operon constructs (containing *FBP1-FLAG*, *FBA1-myc* and *atpB* coding sequences) that were bombarded into cells of strain CC-373, showing regions of the chimeric *FBP1*, *FBA1*, and *atpB* genes amplified by primer sets P1, P2, and P5. White box labeled s, spacer. (B) Genomic PCR analysis of transformants using primer sets P1 to amplify a region within chimeric FBP1 (left panel), P5 to amplify regions of FBA1 and atpB (middle panel), and P2 to amplify chimeric *atpB* (right panel). Genomic DNA was extracted from transformants and from recipient strain CC-373, which served as negative control. pFBP-ts-FBA-ts-atpB was used as positive control for each set of PCR.
